# Adaboost-Based Machine Learning Improved the Modeling Robust and Estimation Accuracy of Pear Leaf Nitrogen Concentration by In-Field VIS-NIR Spectroscopy

**DOI:** 10.3390/s21186260

**Published:** 2021-09-18

**Authors:** Jie Wang, Wei Xue, Xiaojun Shi, Yangchun Xu, Caixia Dong

**Affiliations:** 1 College of Resources and Environment, Southwest University, Chongqing 400716, China; mutouyu@swu.edu.cn (J.W.); shixj@swu.edu.cn (X.S.); 2 College of Resources and Environmental Sciences, Nanjing Agricultural University, Nanjing 210095, China; ycxu@njau.edu.cn; 3 College of Artificial Intelligence, Nanjing Agricultural University, Nanjing 210095, China; xwsky@njau.edu.cn

**Keywords:** mixed cultivars, VIS-NIR spectroscopy, Adaboost, support vector regression, back-propagation neural networks

## Abstract

Different cultivars of pear trees are often planted in one orchard to enhance yield for its gametophytic self-incompatibility. Therefore, an accurate and robust modelling method is needed for the non-destructive determination of leaf nitrogen (N) concentration in pear orchards with mixed cultivars. This study proposes a new technique based on in-field visible-near infrared (VIS-NIR) spectroscopy and the Adaboost algorithm initiated with machine learning methods. The performance was evaluated by estimating leaf N concentration for a total of 1285 samples from different cultivars, growth regions, and tree ages and compared with traditional techniques, including vegetation indices, partial least squares regression, singular support vector regression (SVR) and neural networks (NN). The results demonstrated that the leaf reflectance responded to the leaf nitrogen concentration were more sensitive to the types of cultivars than to the different growing regions and tree ages. Moreover, the AdaBoost.RT-BP had the best accuracy in both the training (R^2^ = 0.96, root mean relative error (RMSE) = 1.03 g kg^−1^) and the test datasets (R^2^ = 0.91, RMSE = 1.29 g kg^−1^), and was the most robust in repeated experiments. This study provides a new insight for monitoring the status of pear trees by the in-field VIS-NIR spectroscopy for better N managements in heterogeneous pear orchards.

## 1. Introduction

Pear has been cultivated in China for at least 3000 years [1,2]. It is currently widely grown over an area of 1.12 × 10^6^ ha [3]. China’s pear production (1.87 × 10^7^ tons) represents 75 percent of the world’s total yield [4]. However, over-fertilization of nitrogen (N) and phosphorus, common in pear orchards of North China [5,6,7], has led to a low N use efficiency and severe environmental degradation, resulting in accelerated soil acidification, salinization and water quality impairment [8,9,10]. For steady growth and increased fruit production, it is necessary to know the timely N status of pear trees, so that orchardists can provide the correct amount of N fertilizer, optimize N use efficiency, and avoid N losses [11,12]. Despite the costly and labor-intensive chemical tissue testing method of leaf N determination, the recent development and improvement of spectroscopy techniques provide a rapid, non-destructive method for linking leaf N concentration and spectral signatures [13,14,15,16,17].

There are two broad approaches for analyzing hyperspectral data set modeling: physically based and empirically based [18,19]. Recently, both types of leaf N retrieval methods have expanded into subcategories and combinations thereof [20], which can be classified into five methods (adapted from Berger et al., 2020).

- Physically based model inversion methods (radiative transfer models, RTMs)
- Parametric regression methods (vegetation indices with narrow spectra)
- Nonparametric regression methods (including linear and nonlinear approaches)
- Alternative data (sun-induced fluorescence)
- Mixed regression methods.

In general, a parametric regression method is defined by narrow spectra and is then linked to the leaf N concentration through a fitting function [21], which is focused on the visible and near-infrared spectral domains (400–900 nm). In addition, the trend that uses the physically based model inversion methods of RTMs, nonlinear nonparametric regression methods of machine learning, and hybrid techniques, increase. As physically based RTMs, Li et al. (2018) modified the PROSPECT model version into an N-PROSPECT by replacing the specific absorption coefficients corresponding from the leaf chlorophylls to the leaf N concentration, which succeeded in retrieving both leaf and canopy N status [22]. However, this approach was restricted to different cultivars. The observed spectra in various field conditions were influenced by extraneous factors, including both leaf structure and the environmental conditions of the workplace [23]. Our previous study has compared the two methods in modeling the leaf nitrogen concentration of pear leaves:

- Nonlinear nonparametric method of partial least squares regression (PLSR), R^2^ = 0.85
- Parametric regression method of difference vegetation indices (DVI), R^2^ = 0.46.

The PLSR is reported as an effective method for dealing with near-infrared reflectance spectra’ high collinearity [18,24,25]. However, leaf N concentrations and spectra collected in-field of pear trees may vary significantly in different cultivars grown in different regions. As a member of the Rosaceae family, pear presents typical gametophytic self-incompatibility. Therefore, different cultivars of pear trees are often cultivated in one pear orchard to enhance yield and quality [26]. The leaf reflectance of different pear cultivars responding to the leaf nitrogen concentrations have still not been characterized. Recently, machine learning regression algorithms (nonparametric regression approaches), apply nonlinear transformations to capture the nonlinear relationships of mixed spectroscopic data with target variables [27]. Support vector regression (SVR) and neural networks (NN) are two of the most widely nonlinear nonparametric methods used for estimating foliage biophysical parameters [28,29,30,31,32]. The Adaptive Boosting (Adaboost) algorithm, proposed by Freund (1997), is one of the most successful recognition algorithms in the field of machine learning. The Adaboost algorithm assumes that a combination of weak learners can be “boosted” into an accurate strong learner, which creates a set of weak learners by maintaining a collection of weights over training data and adjusts them after each weak learning cycle adaptively [33]. Recent research has demonstrated that Adaboost-based machine learnings could achieve high accuracy in modelling with multi-class imbalanced data compared to the regular back-propagation neural networks or the convolutional neural network [34,35]. Adaboost has been applied in ensemble learning due to its excellent classification performance, including image recognition, fruit biochemical parameter estimation, and complex change prediction modelling [36,37,38,39]. 

Based on our previous studies, the objectives of this paper were twofold: (1) to evaluate the effect and relationship of different cultivars, growth regions, and tree ages on pear leaf reflectance; and (2) to apply a highly accurate and robust mixed algorithm for estimating leaf N concentration of different cultivars, growth regions and tree ages in pear orchards.

## 2. Materials and Methods

### 2.1. Study Area

The study was conducted in intensive pear production orchards of four main growing regions in the east, north, southwest, and northwest of China. The location, climate, soil, physical and chemical characteristics, tree age, and yield of sampled orchards are detailed in Table 1. Pear leaves were sampled from five cultivars named ‘Kotobuki shinsui’ (*Pyrus pyrifolia* Nakai), ‘Huangguan’ (*P. bretschneideri* Rehd.), Yali (*P. bretschneideri*), ‘Yuanhuang’ (*P. communis*), and ‘Cuiguan’ (*P. pyrifolia*) in different orchards. Climatic differences (temperature and precipitation) among the eastern, western, northern, and southern regions lead to differences in the cultivars and maturity time. For example, Kotobuki shinsui is mainly cultivated in the southern areas because of its relatively large precipitation demand. Huangguan is widely cultivated in mainland China, but its maturity time depends on the effective accumulated temperature. In Gansu Province, Huangguan pear trees blossom in late April, and the fruit is harvested in late September. However, in Jiangsu province, the tree blossom and fruit harvest of Huangguan take place at least one month earlier than in Gansu. Among the six sampled sites, orchards in Yixing and Pengzhou were relatively young (less than ten years old), and yields were relatively low. Pear trees in orchards of Gaochun, Xuzhou, Xinji, and Jingtai were in the full productive age (over ten years old), and yields were higher than that of young orchards. The application rate of N fertilizers in the six orchards ranged from 0 to 490 kg N ha^−1^. The different N treatments were conducted by considering tree ages, the local soil conditions, and average yields. The N treatments in all the regions were the located fertilization experiments by the modern agricultural industry technology system, with 2–5 replicates of 2–5 trees, each arranged in 2 alternate tree rows during 2015–2016. Fruit yields of different cultivars grown in different regions differed from the cultivar characteristic, local climate, and orchard management. The average yield listed in Table 1 were the average values of different N managements. Because of the experimental fertilization, both N deficiency and over-application of N often took place in the same orchard.

### 2.2. Spectra Collection

Nitrogen concentrations in the middle leaves of new shoots from the external side (east, south, west, and north) of the canopy during the 50–80 days after full bloom (50–80 DAFB) were suggested to assess the tree’s N status [40]. In 2015 and 2016, eight to ten leaves per tree were sampled from different cultivars grown in different regions. All leaf samples were collected from multiple plants and were free of insect or fungal infestation. To obtain the high signal-to-noise ratio of leaf spectra, the in-field leaf spectral measurements were conducted using the ASD FieldSpec 3 spectroscopy (Analytical Spectral Devices, Boulder, CO, USA), the assembly of which attached a leaf clip with the black background and a plant probe with an internal stable light source [40]. The FieldSpec 3 spectroscopy covered wavelengths from 350 nm to 2500 nm, with high spectral resolution and resampling accuracy. Before leaf spectra measurement, the leaf-clip with Teflon white standard should be applied to adjust the maximum reflectance (99.9%) conditions. The leaf clip with the black background was used to collect the leaf spectra through the ratio of leaf reflectance and the standard white reflectance. The adaxial leaf surface should be faced to the plant probe. Two symmetrical points beside the leaf vein were designed to collect the spectra. Final leaf spectra were obtained by the average spectrum of the two points.

### 2.3. Determination of Leaf Nitrogen Concentration

Leaf N concentration of dry mass was determined by the Dumas method using an Elementar Vario Macro CHN analyzer (Elementar Analysensyteme GmbH, Hanau, Germany). The leaves which completed spectra measurements were taken to the laboratory for analysis. The leaf samples were dried in an oven first, at 105 °C for 1 h to de-enzyme and then at 70 °C for 72 h to remove the water. The central vein in the middle of the leaves should be removed. The dried mesophyll was finely ground, mixed, and weighted in the Tin boat for determination with standard acetanilide samples.

### 2.4. Sample Division

Considering the large amount of data used in this paper, we chose the k-fold method to perform the cross-test. It is essential to ensure the distribution uniformity of data in each training and test subsets, consistent with the original data distribution. Therefore, stratified sampling is adopted to select the training set and test set. The 1285 samples were collected and composed of 11 subsets. Two-thirds of each subset was randomly selected as the training set and the rest as the test set. In addition, the stratified random sampling was repeated 20 times to test the uniformity and robustness of the modelling methods.

### 2.5. Modelling Methods

In addition to the new machine learning methods, parametric regression methods and linear nonparametric regression methods were also conducted and compared. The parametric regression models are composed of the leaf N concentration and narrowband indices (difference vegetation index DVI, ratio vegetation index—RVI, and normalized difference vegetation index—NDVI) with the method of Yao et al., 2010 [41]. To simplify the computation and to decrease the collinearity of leaf spectra, the narrowband vegetation indices were read and calculated at intervals of 10 nm within the range of 350–2500 nm. All the obtained DVI, RVI, and NDVI were regressed with the reference leaf N concentration by the linear equation. Next, the best linear model and its sensitive bands will be achieved. The establishment of the linear nonparametric regression method (partial least squares regression) was conducted in MATLAB R2017b (MathWorks, Natick, MA, USA). In addition, we used quadratic loss as the loss function. The regular neural network (NN) is composed of three layers: (1) input layer; (2) hidden layer; and (3) the output layer. NN’s task is to minimize the error between the reference and calculated values by adjusting the layers’ weights. In this study, the neural network had three layers, in which the number of neurons in the input layer is not fixed. We used principal components analysis (PCA) for dimensionality reduction and then used sufficient principal components to explain 99.99% of the variance. The hidden layer has 14 neurons, and the output layer has one neuron. The support vector regression (SVR) is mainly used in the regression analysis, which belongs to a supervised learning algorithm [42,43]. We used an SVR with the radial basis function kernel [44]. In this study, the kernel function of SVR is the Gaussian kernel function. The form is as follows:

(1) $$k\left(x,z\right)=\exp\left(-\frac{{\left|\left|x-z\right|\right|}^{2}}{{2\sigma }^{2}}\right)$$

In this study, Adaboost was adapted to combine with a regression method to realize the final high-precision regression model. The Adaboost algorithm was initiated in NN and SVR regression modelling procedures to improve NN and SVR’s predictive ability. Because the dimensionality of training data is very high (2151), the principal component analysis would reduce the training and test sample’s dimension. Consequently, the component conforms to the condition of AdaBoost, and the computational time, could be saved (see the diagram and detail calculation steps in the Supplementary Materials of Figure S1 and attached formulas). 

Moreover, the randomness of the result produced by NN and SVR would decrease significantly after many Adaboost iterations. As a result, the outcomes corresponding to several independent runs of the mixed method are similar. To test the robustness and stability, the process of the hybrid algorithm is computed with 20 repetitions [45,46,47]. The computational steps of the AdaBoost.RT-BP and Adaboost-SVR can be find in the Supplementary Materials. The NN, SVR, parametric regression methods, and the new machine learning methods, were calculated and completed in MATLAB R2017b (MathWorks, Natick, MA, USA).

The accuracy and precision of different models were evaluated by the coefficient of determination (R^2^) between predicted and chemical-determined N concentrations, and root mean squared error (RMSE). According to the criteria of Saeys et al. (2005), training and test results with an R^2^ value greater than 0.91 are considered to be excellent, whereas R^2^ between 0.82 and 0.90 represents a good prediction [48]. RMSE values of training and test results should be small to approximate the measured value. The equations used to calculate these parameters are as follows:

Coefficient of determination:

(2) $$R^{2}\left(y,\hat{y}\right)=1-\frac{\sum_{i=1}^{m_{\mathrm{samples}}}{\left(y_{i}-{\hat{y}}_{i}\right)}^{2}}{\sum_{i=1}^{m_{\mathrm{samples}}}{\left(y_{i}-\bar{y}\right)}^{2}}$$

In which, $\bar{y}=\frac{1}{n_{\mathrm{samples}}}\overset{n_{\mathrm{samples}}}{\underset{i=1}{\sum }}y_{i}$

Root mean squared error:

(3) $$\mathrm{RMSE}\left(y,\hat{y}\right)=\sqrt{\frac{1}{m_{\mathrm{samples}}}\overset{m_{\mathrm{samples}}}{\underset{i=1}{\sum }}{\left(y_{i}-{\hat{y}}_{i}\right)}^{2}}$$

where $\;y_{i}$ is the true value of number i, ${\hat{y}}_{i}$ is the predicted value of number i.

## 3. Results

### 3.1. Leaf N Concentrationon

Statistics of leaf N concentration of the different sample sites are shown in Table 2. Samples were collected from different years, cultivars, and regions of mainland China. The average leaf N concentrations of Yali and Kotobuki shinsui were 25.3 and 23.7 g kg^−1^, respectively, which were significantly lower than that of other cultivars. Nevertheless, we found no significant difference in the average leaf N concentrations of 5-year Cuiguan trees in Yixing and 8-year Cuiguan trees in Pengzhou. The same tendency was found between the 20-year Huangguan in Xinji and the 17-year Huangguan in Jingtai. Differences in leaf N due to trees’ year and cultivation regions were less than those, due to different cultivars.

### 3.2. Leaf Reflectance Spectra

**Figure 1:**
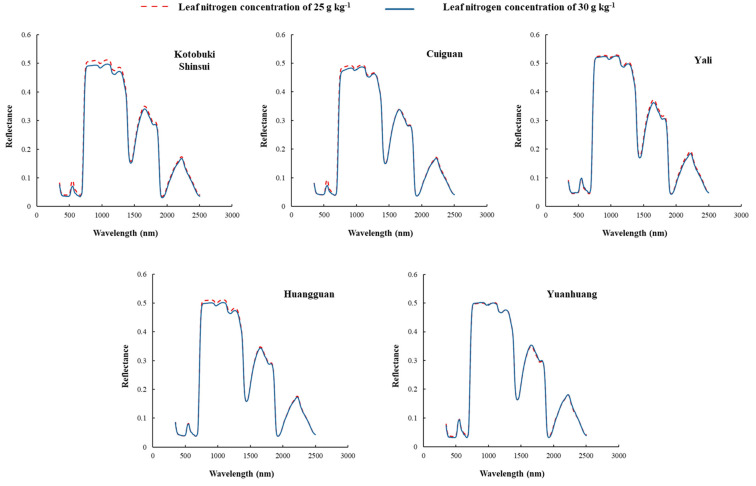
Leaf spectra of five cultivars with two different leaf nitrogen concentrations (25 g kg^−1^ and 30 g kg^−1^). The spectra in visible and near infrared regions of Kotobuki Shinsui and Cuiguan differed with different leaf nitrogen concentrations. In addition, the spectra in near infrared regions of Huangguan differed with different leaf nitrogen concentrations. However, the leaf spectra in all regions of Yali and Yuanhuang were not apparently differed from different leaf nitrogen concentrations.

The leaf reflectance of five pear cultivars with leaf N concentration of 25.0 g kg^−1^ and 30.0 g kg^−1^ were artificially selected to compare the differences induced by the cultivars (Figure 1). The distribution of leaf spectra collected from different cultivars showed the same trait as other foliar spectra. However, the leaf spectra of different cultivars differed at certain bands. In detail, the relationship between spectra and leaf N concentration for each cultivar were significantly different at the same leaf nitrogen concentration difference value. The spectra in visible and near-infrared regions of Kotobuki Shinsui and Cuiguan differed with different leaf nitrogen concentrations. In addition, the spectra in near infrared regions of Huangguan differed with different leaf nitrogen concentrations. However, the leaf spectra in all regions of Yali and Yuanhuang were not apparently differed from different leaf nitrogen concentrations.

**Figure 2:**
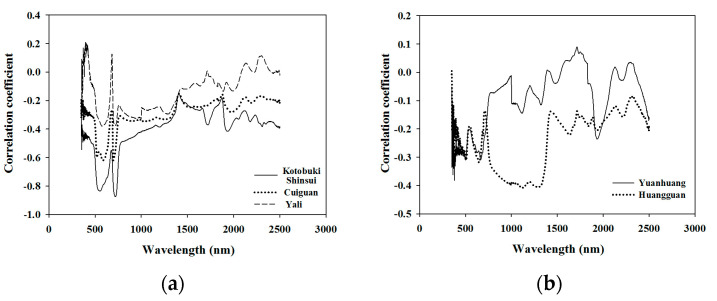
Correlation coefficient between different varieties of leaf nitrogen concentration and the original spectra. The trend of correlation coefficients of Kotobuki shinsui, Cuiguan, and Yali were found to be similar to each other, which showed a higher correlation in the 550 nm (green peak) and 720 nm (red edge). Nevertheless, the trend of correlation coefficient of Huangguan and Yuanhuang were found to be significantly different compared with the three cultivars above.

The correlation coefficients between the leaf N concentration and the leaf spectra of different cultivars, were plotted to better understand inter-cultivar variability for this parameter (Figure 2). The trends of the correlation coefficients of Kotobuki shinsui, Cuiguan, and Yali were found to be similar, with a higher correlation in the 550 nm (green peak) and 720 nm (red edge), but the values of the correlation coefficients in the green peak and the red edge varied from one cultivar to the other (Figure 2a). Nevertheless, the trends in the correlation coefficients of Huangguan and Yuanhuang (Figure 2b) were significantly different to that of the three cultivars in Figure 2a. The correlation coefficient values of Huangguaan at 850 nm to 1350 nm band were higher than those of other wavelengths, while the leaf spectra at wavelengths 670 nm and 1920 nm of Yuanhuang presented high correlation values (Figure 2b). The leaf weight per unit area of different cultivars affected by the same difference value of leaf nitrogen concentration in the supplementary could partially demonstrate that there is a difference in leaf structures between different cultivars (Figure S2).

### 3.3. Modelling Results

The 1285 samples were collected and composed of 11 subsets and two-thirds of each subset was randomly selected as the training set and the rest as the test set (Table 3). The parametric regression models composed of the leaf N concentration and narrowband indices (difference vegetation index DVI, ratio vegetation index—RVI, and normalized difference vegetation index—NDVI) by Yao et al. (2010) were used to identify the bands that resulted in high R^2^ values. Contour maps of R^2^ for the linear relationship between the narrowband indices and the leaf N concentrations of different cultivars were shown in Figure S3.

The R^2^ between leaf N concentration and DVI, RVI, and NDVI ranged from 0.35 to 0.45 in training as well as 0.32 to 0.42 in the test. The wavelengths of 2170 nm and 2160 nm indicated the highest R^2^ between leaf N concentration and DVI, while the wavelengths (1720 nm and 580 nm) resulted in the highest correlation with RVI and NDVI. DVI had the highest R^2^ among the three vegetation indices. Compared with the vegetation indices, PLSR showed a good modelling accuracy during training (R^2^ = 0.85), but the predictive accuracy during the test (R^2^ = 0.76) was relatively lower. Compared with the singular modelling methods of SVR and NN, Adaboost-initiated NN significantly improved the model accuracy in both training and test (Table 4). However, the AdaBoost SVR algorithm performs essentially identically to the standard SVR algorithm (limited improvement of the modelling accuracy in the test subset). The R^2^ of Adaboost combined with NN for a test was above 0.9, which was significantly higher than that of other methods. AdaBoost.RT-BP had advantages over other methods, and fitted with the leaf reflectance and N concentration of different pear cultivars. The five machine learning methods of RMSE ranged from 1.03 to 1.57 g kg^−1^ and 1.29 to 1.78 g kg^−1^, respectively. Similarly, the errors of AdaBoost.RT-BP in both the training and test sets were lower than those of other methods. AdaBoost.RT-BP had the best modelling accuracy in both the training and test sets.

**Figure 3:**
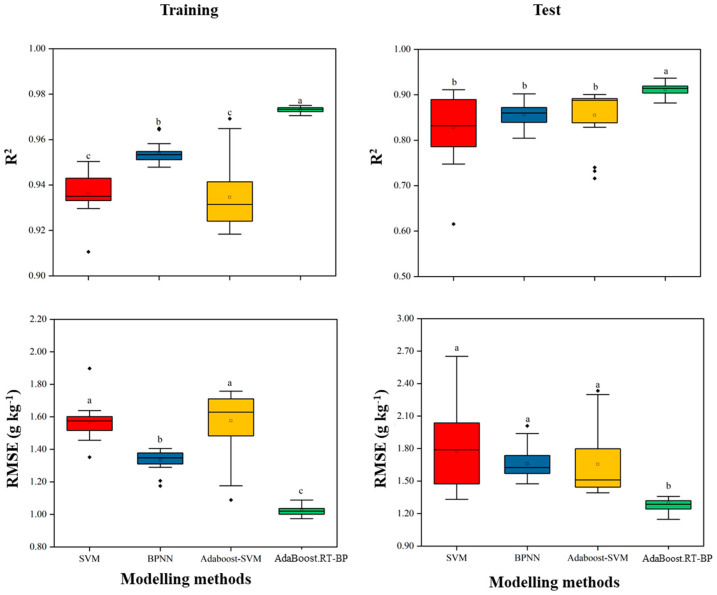
Modelling results of twenty times robust tests by four methods. Adaboost combined with NN outperformed the other modelling methods on the robustness testing. R^2^ is the coefficient of determination of training and test, respectively; RMSE is the root mean relative error. Different letters (a, b and c) indicated the significantly different of modelling methods by the least significant difference (LSD) multiple range test (*p* < 0.05) in SPSS 18.0 software.

To test the stability of the modelling accuracy of the four machine learning methods, 20 random tests were conducted by the stratified random sampling data (Figure 3). A larger interquartile range and the outliers means a relatively bad robustness. In general, NN showed better stability than SVR because of the lower standard deviation in both R^2^ and errors. Compared with the singular modelling methods (SVR, NN), Adaboost initiated in SVR and NN improved the modelling accuracy and significantly reduced the low precision times in both training and test (Figure 3). The robustness of the SVR and the Adaboost-SVR models were not as good as NN. In this study, Adaboost iteratively selected several learner instances by maintaining an adaptive weight distribution, which improved the modelling accuracy and robustness of NN over the training examples. Among the four modelling methods, Adaboost combined with NN (Adaboost.RT-BP) outperformed the others on robustness.

**Figure 4:**
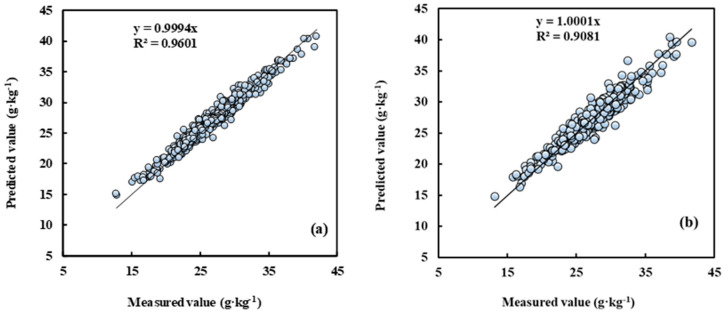
Measured vs. predicted N concentration for training and test by the AdaBoost.RT-BP model. Note: (**a**) is the training set, *n* = 856; (**b**) is the test set, *n* = 429.

The sample set of five cultivars located in different planting regions was randomly split into a training set (*n* = 856) and a test set (*n* = 429), with a split ratio of 2:1. Compared with the other seven modelling methods, the R^2^ of measured leaf N concentration and the predictive value by the AdaBoost.RT-BP model was above 0.9 both in the training and test sets (Figure 4). Accordingly, this model’s root mean square error was less than 1.29 g kg^−1^. The result indicated that the model established by the AdaBoost.RT-BP method satisfies the non-destructive leaf N concentration determination of different cultivars and regions in pear orchards.

## 4. Discussion

### 4.1. Leaf Reflectance Responses to Nitrogen Concentration of Different Cultivars

In the study, we analyzed the relationship between leaf reflectance and N concentrations of different cultivars from different growing regions. The leaf spectral characteristics of different cultivars with different N concentrations were roughly the same, but the leaf reflectance of cultivars affected by the same difference value of leaf nitrogen concentration varied especially in the near-infrared region. In addition, the leaf weight per unit area of different cultivars affected by the same leaf nitrogen concentration further explained that the leaf structure characteristics (leaf thickness) affected by the leaf nitrogen concentration may be the reason that induced the leaf reflectance difference among cultivars (Figure S2). Our result is consistent with the study reported by Li et al. (2018) and Wang et al. (2012) on rice and wheat, who reported that the leaf reflectance affected by different cultivars was more sensitive than that of different growing regions [22,49]. Further analysis of the correlation coefficient between the leaf reflectance and measured leaf N concentration was found to consolidate this result. In addition, the future work should take the leaf picture, determination of the chlorophyll concentration or the leaf thickness to explain the difference induced by the cultivars.

### 4.2. Comparison of Modelling Methods

The wavelengths with the maximum R^2^ response to leaf N concentration were found to be similar in our previous study (2170 nm and 2150 nm), covering a large range of cultivars and nitrogen concentrations. However, the R^2^ is relatively low, and the wavelengths were probably highly correlated according to the result. The modelling accuracy in this study was much lower than that of crops’ N determination by the parametric regression models exploiting limited bands of VIS, red edge, NIR, and SWIR [50]. The maximum R^2^ response to leaf N concentration of wheat was in the region of visible near-infrared spectra [41]. Nevertheless, future work should insist on trying more possible indexes to reduce the amount of input data. The parametric regression models using limited bands were easily influenced by the leaf nitrogen allocation [51]. Recent researchers have demonstrated that leaf N concentration expressed by the leaf area-based measurement was higher correlated to the photosynthetic capacity [52,53]. Nevertheless, some studies have emphasized that vegetation indices using the SWIR regions by the leaf N allocation to protein could improve the modelling accuracy [54]. Coincidentally, our results revealed that leaf N concentration in the pear tree might be allocated more as the non-photosynthetic N (such as proteins and structural N), which were more sensitive to the short-wave infrared regions [19]. In addition, PLSR, which was found optimal in our previous study, including only one cultivar, did not perform well in the present study’s mixed cultivar setting.

Regular NN and SVR have been widely used in regression dealing with high-dimensional data. The modelling performance of NN was superior to the SVR in this study. The trait of nonlinear regression in the SVR modelling procedure is insensitive to random noise [42]. Adaboost is one of the most successful recognition algorithms in machine learning, which is based on the idea that a combination of simple learners (obtained by a weak learner) can perform better than any of the simple learners alone [34]. As a result, Adaboost iteratively selects several learner instances by maintaining an adaptive weight distribution over the training examples, improving the modelling accuracy and robustness of SVR and NN [35]. Compared with single SVR and NN modelling, Adaboost combined with NN can reducing the RMSE in the training and test than the regular NN. However, the AdaBoost SVR algorithm performs essentially identically to the standard SVR algorithm. The experimental results show that Adaboost SVR did have a better effect in the test subset, but the improvement of the modelling accuracy was not that large (Table 4). Wickramaratna et al. (2001) demonstrated that boosting productivity would fall if the underlying learner was a strong regression method (SVR) [55]. Among the machine learning methods, Adaboost combined with NN outperformed the others.

### 4.3. Pear Leaf Nitrogen Determination by the Spectral Method

The published modelling methods listed in Table 5 were evaluated for their ability to predict leaf N concentration of pear trees based on the R^2^ of training and test, mean relative error of the test. Neto et al. (2011) and Yang et al. (2011) used the linear regression method to fit the leaf N concentration of ‘Rocha’ pear trees and the Huanghua pear [12,56]. However, the result of Neto et al. (2011) only demonstrated that SPAD readings ≥33 in leaves sampled at 60–110 DAFB corresponded to optimum leaf N concentration of ≥20 g kg^−1^ dry weight. The linear regression models showed unstable predictive ability during the test (Table 5). The vegetation index (DVI [40]; NDVI [57]) showed the approximate R^2^ value of training and the similar sensitive wavelength of maximum R^2^ in both single cultivars and mixed cultivars (This paper). PLSR can alleviate the high dimensionality of all band spectra input but was weak when dealing with the problem caused by different cultivars (Table 5). The RMSE of modeling by mixed cultivars were found general larger than that of the single cultivars. The R^2^ of the test by the PLSR model with mixed cultivars were 0.72 [58] and 0.76 (this paper), respectively. However, the R^2^ of both training and test of the PLSR model with a single cultivar were above 0.85. Interestingly, the R^2^ of NN showed the opposite result compared to the PLSR. The R^2^ in the test sets by the NN and AdaBoost.RT-BP models with mixed cultivars were 0.85 and 0.92 (this paper), respectively. However, the R^2^ of training and test of the NN model with a single cultivar were 0.89 and 0.67 [40]. Consequently, PLSR was indicated for modelling a single cultivar, and the NN was more suitable for modelling mixed cultivars.

## 5. Conclusions

In this study, machine learning methods were applied to modeling the determination of leaf nitrogen concentration in pear orchards with mixed cultivars by the in-field visible-near infrared spectroscopy. Results showed that the effect of different cultivars on leaf reflectance of pears was greater than that of different growing regions and tree ages. In addition, among the modelling methods analyzed, the AdaBoost.RT-BP performed the best in accuracy and robustness in both training and test sets. The results from this study provide a new method to assess pear trees’ N status for better N managements in pear orchards with mixed cultivars.

## Figures and Tables

**Table 1:** Main characteristics of the sampling areas.

	Jiangsu			Hebei	Gansu	Sichuan
	Gaochun	Yixing	Xuzhou	Xinji	Jingtai	Pengzhou
Location	32.27 N, 118.95 E	31.35 N, 119.74 E	34.26 N, 117.19 E	37.92 N, 115.22 E	37.21 N, 104.06 E	31.03 N, 103.76 E
Annual mean temperature (°C)	15.9	15.7	14.5	12.5	9.1	15.7
Annual mean precipitation (mm)	1157	1177	853	488	186	933
Climate type	subtropical monsoon climate	subtropical monsoon climate	temperate monsoon climate	temperate monsoon climate	temperate continental climate	subtropical monsoon climate
main soil texture	clay loamy	clay loamy	brown soil	sandy soil	sierozem	clay loamy
Soil pH	6.80	6.39	7.78	7.49	8.07	7.51
Soil organic matter (g kg^−1^)	17.07	15.65	9.5	21.6	12.44	7.42
Soil available N (mg kg^−1^)	69.37	21.15	74.97	33.3	62.93	63.35
Soil available P (mg kg^−1^)	48.18	18.61	70.20	31.1	68.97	58.46
Soil available K (mg kg^−1^)	146.3	127.8	182.0	119.0	157.05	211.18
N rate (kg N ha^−1^)	0, 165, 330, 490	0, 66, 132, 198	180–350	150–390	220, 462	110, 235
Planting density (m)	4 × 4	4 × 3	4 × 4	4 × 4	4 × 4	4 × 3
Cultivars	Kotobuki shinsui	Cuiguan	Huangguan, Yuanhuang	Huangguan, Yali, Yuanhuang	Huangguan	Cuiguan
Tree age (years)	12	5	14	20	17	8
Average Yield (kg ha^−1^)	16,500	2475	47,800 (Huangguan) 45,050 (Yuanhuang)	45,000 (Huangguan) 41,250 (Yuanhuang) 52,500 (Yali)	50,600	12,800

**Table 2:** Statistics of leaf nitrogen concentrations of different cultivars.

Year	Sample Subset	Cultivar	Sample Number	Leaf Nitrogen Concentration (g kg^−1^)		
				Min.	Max.	Mean ^†^
2015	Gaochun	Kotobuki Shinsui	160	12.7	35.7	23.7 ± 5.0b
	Yixing	Cuiguan	200	21.0	42.0	29.6 ± 4.5a
	Xinji	Huangguan	189	22.5	36.9	29.5 ± 2.7a
	Xinji	Yali	193	16.7	29.7	23.6 ± 2.3b
	Xinji	Yuanhuang	197	21.4	35.6	26.7 ± 2.6ab
2016	Pengzhou	Cuiguan	96	21.7	38.5	28.0 ± 3.6a
	Xuzhou	Huangguan	40	25.5	32.3	28.8 ± 1.5a
	Xuzhou	Yuanhuang	46	22.1	32.2	26.9 ± 2.6ab
	Xinji	Huangguan	49	26.3	33.0	28.0 ± 1.5a
	Xinji	Yali	35	22.4	31.1	25.9 ± 2.1b
	Jingtai	Huangguan	80	22.6	32.4	28.5 ± 2.3a

^†^ Values were expressed as mean ± SD. Different letters indicated the significantly different of groups by the least significant difference (LSD) multiple range test (*p* < 0.05) in SPSS 18.0 software.

**Table 3:** Statistical values of N concentration of pear leaves used for training and test.

Data Sets	Sample No.	Leaf Nitrogen Concentration (g kg^−1^)		
		Min.	Max.	Mean ^††^
All	1285	12.74	41.99	26.98 ± 3.96
Training	856	12.74	41.99	26.93 ± 3.86
Test	429	13.11	41.74	27.03 ± 4.17

^††^ Values were expressed as mean ± SD.

**Table 4:** Coefficient of determination and errors of training and test of nine modelling methods.

Modelling Methods ^†††^	Training		Test		Wavelength of Max. R^2^
	R^2^	RMSE (g kg^−1^)	R^2^	RMSE (g kg^−1^)	
DVI	0.45	3.77	0.42	4.55	2170 nm, 2160 nm
RVI	0.40	5.98	0.38	6.15	1720 nm, 580 nm
NDVI	0.35	7.06	0.32	7.48	1720 nm, 580 nm
PLSR	0.85	2.07	0.76	3.46	——
SVR	0.94	1.57	0.83	1.78	——
NN	0.95	1.33	0.86	1.66	——
Adaboost-SVR	0.93	1.58	0.85	1.66	——
AdaBoost.RT-BP	0.96	1.03	0.91	1.29	——

^†††^ DVI, RVI, NDVI, PLSR, SVR and NN represent difference vegetation indexes, ratio vegetation indexes, normalized differential vegetation indexes, partial least squares regression, support vector regression and neural networks, respectively. R^2^ is the coefficient of determination; RMSE is the root mean squared errors.

**Table 5:** The coefficients of determination (R^2^) of training and test, mean relative error of test (MRE) for estimating nitrogen concentration of pear leaves in comparative studies.

Method ^†††^	Pear Cultivars	Training	Test		Reference
		R^2^	R^2^	RMSE (g kg^−1^)	
Linear regression	Rocha, Huanghua	0.87	0.54 to 0.99	No detail data	Neto et al., 2011; Yang et al., 2011
PLSR	Cuiguan, Huangguan	0.90	0.72	2.95	Wang et al., 2014
Vegetation index	Kotobuki shinsui, Red-blush	0.46–0.67	0.41–0.51	3.0–3.35	Wang et al., 2017; Perry et al., 2018
PLSR	Kotobuki shinsui	0.86	0.85	1.50	Wang et al., 2017
NN	Kotobuki shinsui	0.89	0.67	1.70	Wang et al., 2017
Vegetation index	Mixed cultivars	0.45	0.42	4.55	This paper
PLSR	Mixed cultivars	0.85	0.76	3.46	This paper
NN	Mixed cultivars	0.95	0.85	1.66	This paper
AdaBoost.RT-BP	Mixed cultivars	0.97	0.92	1.29	This paper

^†††^ DSI, PLSR, and NN represent difference spectral index, partial least squares regression, and neural networks, respectively.

## Supplementary Materials

The following are available online at https://www.mdpi.com/article/10.3390/s21186260/s1, Figure S1: The schematic illustration of Adaboost analysis, Attached formulas: The computational steps of the AdaBoost.RT-BP and Adaboost-SVR, Figure S2: the leaf weight per unit area of different cultivars affected by the same leaf nitrogen concentration, Figure S3: Contour maps of R^2^ for the linear relationship between the narrowband indices (DVI, RVI and NDVI) and the leaf N concentration of different cultivars.
