# Supplementary material for: Adaboost-Based Machine Learning Improved the Modeling Robust and Estimation Accuracy of Pear Leaf Nitrogen Concentration by In-Field VIS-NIR Spectroscopy
Source: Sensors (Basel). 2021 Sep 18;21(18):6260. doi: 10.3390/s21186260 (PMC8473462; doi:10.3390/s21186260)
Supplement: Supplementary file 1 [file sensors-21-06260-s001.zip › sensors-1324696-supplementary.pdf]

Supplementary materials

Adaboost-Based Machine Learning Improved the Modeling Robust and Estimation Accuracy of Pear Leaf Nitrogen Concentration by In-Field VIS-NIR Spectroscopy

Jie Wang [1,2], Wei Xue [3], Xiaojun Shi [1], Yangchun Xu [2] and Caixia Dong [2,*]

[1] College of Resources and Environment, Southwest University, Chongqing 400716, China; mutouyu@swu.edu.cn (J.W.); shixj@swu.edu.cn (X.S.)
[2] College of Resources and Environmental Sciences, Nanjing Agricultural University, Nanjing 210095, China; ycxu@njau.edu.cn (Y.X.); cxdong@njau.edu.cn (C.D.)
[3] College of Artificial Intelligence, Nanjing Agricultural University, Nanjing 210095, China; xwsky@njau.edu.cn (W.X.)
* Correspondence: cxdong@njau.edu.cn (C.D.)

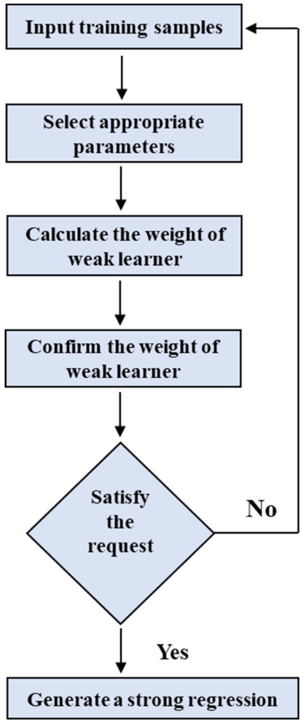

Figure S1. The schematic illustration of Adaboost analysis. The training sample's dimension would be reduced by the principal component analysis. Then, we input the training samples into the calculation procedure. Proper parameters of NN and SVR should be selected appropriately when initial with Adaboost. The weight of weak learners would be calculated. If the generation of weak learners could satisfy the request, the calculation will finish. If not, the calculation will be repeated.

Attached formulas:

The computational steps of the AdaBoost.RT-BP and Adaboost-SVR are explained as follows:

AdaBoost.RT-BP algorithm.

Step 1 Network initialization:

Selection of Training samples data $S = \{(x_1, y_1),(x_2, y_2), \dots, (x_m, y_m)\}$, where x_i is a training instance.

Set the number of iteration ($t = 1$), for later applying to step 4

Initialize the weight

$$\left[D_t\ (i) = \frac{1}{\mathrm{m}}\right], i = 1, 2, \dots, m.$$

m is the number of training samples.

Set the initial error rate $\varepsilon_t = 0$.

According to the input sample's different dimensions, the number of neurons in the corresponding input layer will differ.

Initialize the threshold value of the maximum allowable error $\phi = 0.1$.

Step 2 The dimension reduction of the training sample set is calculated by principal component analysis.

Step 3 Training of NN weak learners:

Different types of BP weak predictors are constructed by selecting different functions.

for (t = 1; t <= T; t++) :

Establish the regression models: $\mathrm{g}_t\ (x) \rightarrow \mathrm{y}$.

$\varepsilon_t = 0$

for(i =1;i<=m;i++):

If $\left|\frac{\mathrm{g}_t\ (x_i) - \mathrm{y}_i}{\mathrm{y}_i}\right| > \phi$:

$\varepsilon_t = \varepsilon_t + D_\mathrm{t}(i)$

$\beta_t = \varepsilon_t^2$

update the weight:

for(i =1; i<=m; i++):

$$D_{t+1}(i) = \frac{D_t(i)}{\mathrm{B_t}} \times \begin{cases} \beta_t, & \left|\frac{\mathrm{g}_t\ (x_i)\ -\ \mathrm{y}_i}{\mathrm{y}_i}\right| \le \phi \\ 1, & \text{otherwise} \end{cases}$$

D_t is the weight of each sample, B_t is the normalization factor that makes the sum of weights equal to 1.

Step 4 Output the strong predictive function:

$$g_{out}(x) = \frac{\sum_{t=1}^{T}\left\{\left(\log\frac{1}{\beta_t}\right)\cdot g_t(x)\right\}}{\sum_{t=1}^{T}\left(\log\frac{1}{\beta_t}\right)}$$

AdaBoost-SVR algorithm

Step 1 Selection of training samples data $S = \{(x_1, y_1),(x_2, y_2), \ldots, (x_m, y_m)\}$, where x_i is a training instance.

Given the parameters of the SVR and the maximum number of iterations *T*. $D_t(i)$ represents the weight of the *i*th sample after learning *t* times from SVR. The threshold value of the maximum allowable error is ϕ =0.1.

Step 2 The training sample set of dimension reduction is obtained by principal component analysis.

Step 3 The initial weight of all samples is $D_t(i) = \frac{1}{m}$, *m* is the number of training samples.

Step 4 Training of SVR weak learners:

For (t = 1; t < =T; t++) :

Set the distribution of weights as $p_i = \frac{D_t(i)}{\sum_{j=1}^{m} D_t(j)}, i = 1,2, \ldots, m$

The regression function $h_t(x_i)$ was obtained by using SVR as a weak learning machine:

$$h_t:\ x \to (x_1, x_2, x_3, \ldots, x_m)$$

Compute the error ε_t:

$$\varepsilon_t = \sum_{i=1}^{m} p_i \times \begin{cases} |h_t(x_i) - y_i|, & if|h_t(x_i) - y_i| \geq \phi \\ 0, & if|h_t(x_i) - y_i| < \phi \end{cases}$$

Compute the weight α_t based on the error ε_t:

$$\alpha_t = \frac{1}{2}\ln\left(\frac{1-\varepsilon_t}{\varepsilon_t}\right)$$

Update the sample weights:

For (i = 1; i < =m; i++):

$$D_{t+1}(i) = D_t(i) \times \begin{cases} exp(-\alpha_t), & if|h_t(x_i) - y_i| < \phi \\ exp(\alpha_t), & if|h_t(x_i) - y_i| \geq \phi \end{cases}$$

Step 5 The final regression function is:

$$H(x) = \frac{\sum_{t=1}^{T} \alpha_t h_t(x)}{\sum_{t=1}^{T} \alpha_t}$$

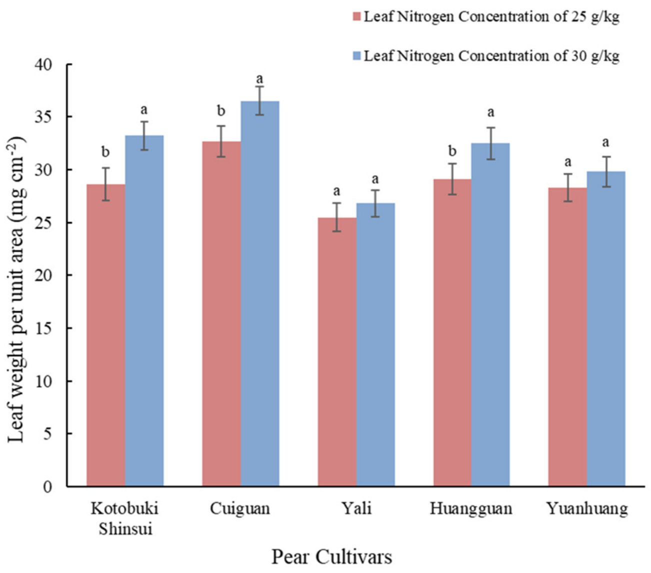

Figure S2. the leaf weight per unit area of different cultivars affected by the same leaf nitrogen concentration. Column with error bars represented the average values of leaves weight per unit area, which calculated by the ratio of leaf weight to the leaf area. Different letters above the error bars represented the significant differences (P < 0.05; Duncan's test) affected by different leaf nitrogen concentrations. The leaf areas were obtained by the Image-Pro Plus 6.0 software (Media Cybernetics, Silver Spring, MD, USA). Leaf images were collected with a digital camera (D700, Nikon, Inc., Japan) by placed them on A4 paper.

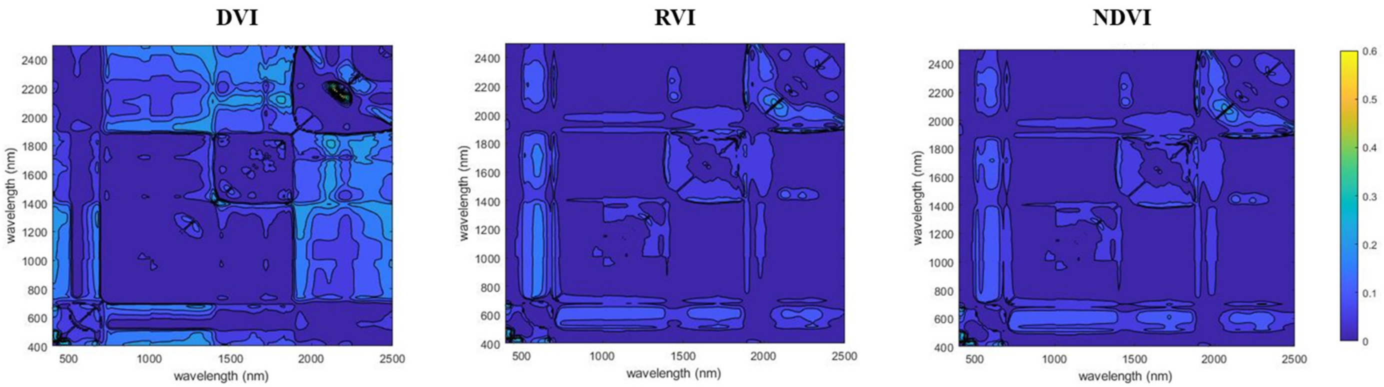

Figure S3. Contour maps of R^2 for the linear relationship between the narrowband indices (DVI, RVI and NDVI) and the leaf N concentration of different cultivars.
